# Flies and Infection

**Published:** 1898-10-29

**Authors:** 


### FLIES AND INFECTION.

A writer in the New York *Medical Record* draws attention to the diminishing use of the old-fashioned chloride of lime as a disinfectant, and urges that it should be liberally employed in all latrines. In this perhaps there is nothing very new. But the reason he gives for preferring this to other disinfectants is an interesting one. He says, "Ten or fifteen pounds thrown into a latrine each day will thoroughly disinfect the excreta, and at the same time banish every fly from the immediate vicinity." The distribution of infection, especially that of cholera and typhoid fever, through the agency of flies is now so well recognised, that the power of keeping such insects away becomes a very important desideratum in any disinfectant which is to be used for latrines in military practice, and for the various dry earth and midden systems which are met with in civil life, and if it is true that chloride of lime, used with some liberality, will keep flies from settling on the excreta, this chemical may possibly be found much more practically useful than other disinfectants, even though their direct bactericidal power may be greater. The writer to whom we refer asks whether anyone has "ever seen or heard of a fly that would visit a sink where the odour of chloride of lime pervaded the atmosphere?" That is just the question.

